# Publisher Correction: The potential for coral reef restoration to mitigate coastal flooding as sea levels rise

**DOI:** 10.1038/s41467-024-49331-9

**Published:** 2024-06-10

**Authors:** Lauren T. Toth, Curt D. Storlazzi, Ilsa B. Kuffner, Ellen Quataert, Johan Reyns, Robert McCall, Anastasios Stathakopoulos, Zandy Hillis-Starr, Nathaniel Hanna Holloway, Kristen A. Ewen, Clayton G. Pollock, Tessa Code, Richard B. Aronson

**Affiliations:** 1 U.S. Geological Survey, St. Petersburg Coastal and Marine Science Center, St. Petersburg, FL USA; 2 U.S. Geological Survey, Pacific Coastal and Marine Science Center, Santa Cruz, CA USA; 3 Deltares, Delft, Netherlands; 4 IHE Delft Institute for Water Education, Delft, Netherlands; 5 National Park Service, Christiansted, VI USA; 6 Florida Institute of Technology, Department of Ocean Engineering and Marine Sciences, Melbourne, FL USA

**Keywords:** Physical oceanography, Restoration ecology, Natural hazards, Climate-change ecology, Climate-change impacts

Correction to: *Nature Communications* 10.1038/s41467-023-37858-2, published online 21 April 2023

The original version of the Peer Review File associated with this Article was related to a different Article. This has been corrected in the HTML version of the article.

### Supplementary information

Peer Review File

