# Supplementary material for: The potential for coral reef restoration to mitigate coastal flooding as sea levels rise
Source: Nat Commun. 2023 Apr 21;14:2313. doi: 10.1038/s41467-023-37858-2 (PMC10121583; doi:10.1038/s41467-023-37858-2)
Supplement: Supplementary file 1 — Supplementary Information [file 41467_2023_37858_MOESM1_ESM.docx]

**Supplementary Information**

for

**A window of opportunity for coral-reef restoration to mitigate coastal flooding as sea levels rise**

Lauren T. Toth^1*^, Curt D. Storlazzi^2^, Elizabeth M. Whitcher^3^, Ilsa B. Kuffner^1^, Ellen Quataert^4^, Johan Reyns^4,5^, Robert McCall^4^, Anastasios Stathakopoulos^1^, Zandy Hillis-Starr^6,7†^, Nathaniel Hanna Holloway^6^, Kristen A. Ewen^6^, Clayton G. Pollock^6^, Tessa Code^6^, Richard B. Aronson^3^

*^1^U.S. Geological Survey, St. Petersburg Coastal and Marine Science Center, St. Petersburg, FL, U.S.A.*

*^2^U.S. Geological Survey, Pacific Coastal and Marine Science Center, Santa Cruz, CA, U.S.A.*

*^3^Florida Institute of Technology, Department of Ocean Engineering and Marine Sciences, Melbourne, FL, U.S.A.*

*^4^Deltares, Delft, Netherlands*

*^5^IHE Delft Institute for Water Education, Delft, Netherlands*

*^6^National Park Service, 2100 Church St. #100, Christiansted, VI 00820*

*^7^46 West 3rd Street, New Castle, DE 19720*

**Data Availability:** All data used in this study are available in USGS Data Releases (reef-survey data: <https://doi.org/10.5066/P97YB2YF>; coral-growth data: <https://doi.org/10.5066/P94BOI9T>; hydrodynamic data: <https://doi.org/10.5066/P947RPG4>).

***Any use of trade, firm, or product names is for descriptive purposes only and does not imply endorsement by the U.S. Government.***

**Table S1**. Carbonate Budgets of the 54 sites analyzed around Buck Island Reef National Monument. Sites highlighted in green showed positive net carbonate production/accretion.

| **Sector** | **Habitat** | **Site** | **Latitude** | **Longitude** | **Coral Cover**  (%) | **Gross Production**  (kg CaCO_3_ m^-2^ y^-1^) | **Bioerosion**  (kg CaCO_3_ m^-2^ y^-1^) | **Net Production**  (kg CaCO_3_ m^-2^ y^-1^) | **Accretion**  (mm y^-1^) | **Δ Elevation 2100** (m) |
| --- | --- | --- | --- | --- | --- | --- | --- | --- | --- | --- |
| North | Fore Reef | N1 | 17.7950 | -64.6280 | 1.33 | 0.46 | 3.71 | -3.25 | -1.94 | -16.33 |
|  |  | N2 | 17.7946 | -64.6275 | 3.07 | 0.40 | 6.16 | -5.76 | -3.45 | -28.98 |
|  |  | N3 | 17.7941 | -64.6267 | 2.44 | 0.25 | 9.11 | -8.86 | -5.31 | -44.57 |
|  |  | N4 | 17.7940 | -64.6263 | 0.56 | 0.09 | 5.94 | -5.86 | -3.51 | -29.45 |
|  |  | N5 | 17.7930 | -64.6247 | 0.27 | 0.00 | 5.71 | -5.70 | -3.42 | -28.69 |
|  |  | N6 | 17.7918 | -64.6187 | 4.30 | 0.71 | 3.69 | -2.99 | -1.79 | -15.01 |
|  |  | E1 | 17.7904 | -64.6135 | 1.20 | 0.15 | 2.14 | -1.99 | -1.19 | -9.99 |
|  |  | E2 | 17.7902 | -64.6118 | 3.22 | 0.41 | 3.49 | -3.09 | -1.85 | -15.54 |
|  |  | E3 | 17.7889 | -64.6089 | 0.56 | 0.01 | 4.11 | -4.10 | -2.46 | -20.64 |
|  | Reef Crest | N1 | 17.7949 | -64.6281 | 1.60 | 0.02 | 4.50 | -4.48 | -2.68 | -22.54 |
|  |  | N2 | 17.7944 | -64.6276 | 5.09 | 0.12 | 5.72 | -5.60 | -3.35 | -28.17 |
|  |  | N3 | 17.7940 | -64.6268 | 2.26 | 0.09 | 4.49 | -4.40 | -2.64 | -22.14 |
|  |  | N4 | 17.7939 | -64.6264 | 4.97 | 0.06 | 5.36 | -5.30 | -3.17 | -26.65 |
|  |  | N5 | 17.7929 | -64.6247 | 2.91 | 0.20 | 2.70 | -2.50 | -1.49 | -12.56 |
|  |  | N6 | 17.7917 | -64.6186 | 7.07 | 0.68 | 2.76 | -2.08 | -1.25 | -10.47 |
|  |  | E1 | 17.7904 | -64.6134 | 5.44 | 0.15 | 2.00 | -1.85 | -1.11 | -9.31 |
|  |  | E2 | 17.7901 | -64.6119 | 12.78 | 1.44 | 1.98 | -0.54 | -0.32 | -2.71 |
|  |  | E3 | 17.7888 | -64.6089 | 12.97 | 2.25 | 5.50 | -3.24 | -1.94 | -16.31 |
|  | Back Reef | N1 | 17.7941 | -64.6282 | 5.89 | 0.74 | 4.87 | -4.14 | -2.48 | -20.81 |
|  |  | N2 | 17.7939 | -64.6277 | 2.77 | 0.28 | 4.12 | -3.84 | -2.30 | -19.30 |
|  |  | N3 | 17.7937 | -64.6270 | 1.67 | 0.23 | 2.68 | -2.45 | -1.47 | -12.35 |
|  |  | N4 | 17.7935 | -64.6265 | 13.52 | 1.59 | 5.25 | -3.66 | -2.19 | -18.41 |
|  |  | N5 | 17.7925 | -64.6244 | 19.47 | 3.93 | 3.14 | 0.79 | 1.18 | 9.91 |
|  |  | N6 | 17.7912 | -64.6188 | 0.21 | 0.00 | 5.51 | -5.51 | -3.30 | -27.71 |
|  |  | E1 | 17.7902 | -64.6133 | 3.07 | 0.48 | 2.50 | -2.02 | -1.21 | -10.16 |
|  |  | E2 | 17.7899 | -64.6118 | 12.92 | 1.79 | 4.07 | -2.28 | -1.37 | -11.47 |
|  |  | E3 | 17.7881 | -64.6091 | 1.58 | 0.18 | 4.24 | -4.07 | -2.44 | -20.45 |
| South | Fore Reef | S1 | 17.7857 | -64.6114 | 5.45 | 1.04 | 6.66 | -5.62 | -3.37 | -28.29 |
|  |  | S2 | 17.7845 | -64.6146 | 6.40 | 1.57 | 6.62 | -5.04 | -3.02 | -25.37 |
|  |  | S3 | 17.7839 | -64.6160 | 6.33 | 2.00 | 4.56 | -2.55 | -1.53 | -12.85 |
|  |  | S4 | 17.7836 | -64.6171 | 3.39 | 0.88 | 3.61 | -2.73 | -1.63 | -13.73 |
|  |  | S5 | 17.7834 | -64.6197 | 2.27 | 0.58 | 4.60 | -4.01 | -2.40 | -20.19 |
|  |  | S6 | 17.7834 | -64.6200 | 8.00 | 2.45 | 3.02 | -0.58 | -0.34 | -2.90 |
| **Sector** | **Habitat** | **Site** | **Latitude** | **Longitude** | **Coral Cover**  (%) | **Gross Production**  (kg CaCO_3_ m^-2^ y^-1^) | **Bioerosion**  (kg CaCO_3_ m^-2^ y^-1^) | **Net Production**  (kg CaCO_3_ m^-2^ y^-1^) | **Accretion**  (mm y^-1^) | **Δ Elevation 2100** (m) |
| South | Fore Reef | E4 | 17.7872 | -64.6091 | 1.58 | 0.21 | 3.49 | -3.28 | -1.96 | -16.49 |
|  |  | E5 | 17.7869 | -64.6094 | 19.52 | 5.93 | 4.31 | 1.63 | 2.13 | 17.90 |
|  |  | E6 | 17.7866 | -64.6097 | 2.40 | 0.62 | 3.56 | -2.94 | -1.76 | -14.80 |
|  | Reef Crest | S1 | 17.7853 | -64.6115 | 36.00 | 10.32 | 5.47 | 4.85 | 6.21 | 52.15 |
|  |  | S2 | 17.7841 | -64.6143 | 16.56 | 2.78 | 2.35 | 0.43 | 0.66 | 5.53 |
|  |  | S3 | 17.7835 | -64.6158 | 16.48 | 4.17 | 6.22 | -2.05 | -1.23 | -10.32 |
|  |  | S4 | 17.7832 | -64.6169 | 32.12 | 8.42 | 3.45 | 4.97 | 6.08 | 51.04 |
|  |  | S5 | 17.7830 | -64.6198 | 14.06 | 1.88 | 5.27 | -3.40 | -2.03 | -17.08 |
|  |  | S6 | 17.7828 | -64.6201 | 8.11 | 1.10 | 4.14 | -3.04 | -1.82 | -15.29 |
|  |  | E4 | 17.7867 | -64.6090 | 4.00 | 1.29 | 3.65 | -2.36 | -1.41 | -11.86 |
|  |  | E5 | 17.7865 | -64.6093 | 3.15 | 0.45 | 4.00 | -3.55 | -2.13 | -17.88 |
|  |  | E6 | 17.7864 | -64.6096 | 3.47 | 0.53 | 6.24 | -5.71 | -3.42 | -28.74 |
|  | Back Reef | S1 | 17.7857 | -64.6114 | 6.95 | 0.97 | 4.40 | -3.43 | -2.05 | -17.25 |
|  |  | S2 | 17.7845 | -64.6146 | 4.89 | 0.76 | 1.40 | -0.64 | -0.38 | -3.23 |
|  |  | S3 | 17.7839 | -64.6160 | 3.24 | 0.99 | 1.50 | -0.51 | -0.30 | -2.56 |
|  |  | S4 | 17.7836 | -64.6171 | 3.76 | 0.49 | 0.88 | -0.39 | -0.23 | -1.95 |
|  |  | S5 | 17.7834 | -64.6197 | 0.62 | 0.07 | 2.88 | -2.81 | -1.68 | -14.12 |
|  |  | S6 | 17.7834 | -64.6200 | 1.85 | 0.22 | 3.21 | -2.99 | -1.79 | -15.04 |
|  |  | E4 | 17.7872 | -64.6091 | 4.44 | 0.86 | 2.58 | -1.71 | -1.03 | -8.62 |
|  |  | E5 | 17.7869 | -64.6094 | 8.67 | 1.09 | 5.31 | -4.23 | -2.53 | -21.26 |
|  |  | E6 | 17.7866 | -64.6097 | 3.56 | 0.39 | 4.94 | -4.54 | -2.72 | -22.86 |

**Table S2.** Coral-reef carbonate budgets at Buck Island Reef National Monument. Average gross carbonate production (kg CaCO_3_ m^-2^ y^-1^; values in bold), bioerosion, net carbonate production, accretion, and projected elevation-change by 2100 C.E. for each habitat in the North and South sectors of the reef (“zones”) are presented, along with the ranges (in parentheses) across the nine sites in each zone and the overall mean (± standard error) across all 54 sites.

| **Sector** | **Habitat** | **Gross Production**  (kg CaCO_3_ m^-2^ y^-1^) | **Bioerosion**  (kg CaCO_3_ m^-2^ y^-1^) | **Net Production**  (kg CaCO_3_ m^-2^ y^-1^) | **Reef-accretion potential**  (mm y^-1^) | **Δ Elevation 2100** (m) |
| --- | --- | --- | --- | --- | --- | --- |
| North | Fore Reef | **0.28**  (<0.01 to 0.71) | **-4.90**  (-2.14 to -9.11) | **-4.62**  (-8.86 to -1.99) | **-2.68**  (-5.31 to -1.19) | **-0.23**  (-0.45 to -0.10) |
|  | Reef Crest | **0.56**  (0.02 to 2.25) | **-3.89**  (-1.98 to -5.72) | **-3.33**  (-5.60 to -0.54) | **-2.00**  (-3.35 to -0.32) | **-0.17**  (-0.28 to -0.03) |
|  | Back Reef | **1.02**  (<0.01 to 3.93) | **-4.04**  (-2.50 to -5.51) | **-3.01**  (-5.51 to 0.79) | **-1.72**  (-3.30 to +1.18) | **-0.15**  (-0.28 to +0.10) |
| South | Fore Reef | **1.70**  (0.21 to 5.93) | **-4.49**  (-3.02 to -6.66) | **-2.79**  (-5.62 to +1.63) | **-1.54**  (-3.37 to +2.13) | **-0.13**  (-0.28 to +0.18) |
|  | Reef Crest | **3.44**  (0.45 to 10.32) | **-4.53**  (-2.35 to -6.24) | **-1.10**  (-5.71 to +4.97) | **0.10**  (-3.42 to +6.21) | **+0.01**  (-0.29 to +0.52) |
|  | Back Reef | **0.65**  (0.07 to 1.09) | **-3.01**  (-0.88 to -5.31) | **-2.36**  (-4.54 to -0.39) | **-1.41**  (-2.72 to -0.23) | **-0.12**  (-0.23 to -0.02) |
| Overall |  | **1.27**  (± 0.27) | **-4.14**  (± 0.21) | **-2.87**  (± 0.33) | **-1.56**  (± 0.27) | **-0.13**  (± 0.02) |

**Table S3.** Summary (mean ± standard error) of estimated bioerosion (kg CaCO_3_ m^-2^ y^-1^) in each reef zone by the different parrotfish species listed from left to right in order of greatest mean bioerosion rates throughout Buck Island Reef National Monument.

| **Sector** | **Habitat** | ***Sparisoma viride*** | ***Scarus***  ***vetula*** | ***Sparisoma aurofrenatum*** | ***Sparisoma rubripinne*** | ***Scarus***  ***iserti*** | ***Sparisoma chryosterum*** | ***Scarus taeniopterus*** |
| --- | --- | --- | --- | --- | --- | --- | --- | --- |
| North | Fore Reef | 2.17±0.51 | 0.44±0.07 | 0.74±0.08 | 0.96±0.23 | 0.03±0.01 | 0.03±0.03 | <0.01±<0.01 |
|  | Reef Crest | 2.39±0.47 | 0.46±0.07 | 0.36±0.12 | 0.16±0.08 | 0.01±<0.01 | *Not observed* | <0.01±<0.01 |
|  | Back Reef | 2.14±0.23 | 0.74±0.12 | 0.49±0.10 | 0.05±0.03 | 0.11±0.03 | *Not observed* | <0.01±<0.01 |
| South | Fore Reef | 2.97±0.46 | 0.30±0.09 | 0.46±0.08 | 0.08±0.04 | 0.01±<0.01 | 0.11±0.07 | 0.01±<0.01 |
|  | Reef Crest | 2.74±0.40 | 0.42±0.06 | 0.42±0.07 | 0.11±0.09 | 0.01±<0.01 | 0.05±0.03 | 0.01±0.01 |
|  | Back Reef | 1.49±0.43 | 0.49±0.15 | 0.38±0.13 | 0.07±0.04 | 0.04±0.01 | *Not observed* | 0.03±0.01 |

**Table S4**. Results of SIMPER analyses comparing taxa contributing most significantly (95% cumulative contribution to dissimilarity) to gross carbonate production of each reef zone at Buck Island Re. The results include the mean and standard deviation (SD) of the percent contribution of each taxon to the dissimilarity between zones, the ratio of the mean to the standard deviation, the average gross carbonate production (kg CaCO_3_ m^-2^ y^-1^) of each taxon in each zone, and the cumulative percent contribution of each taxon to the dissimilarity (% Contribution).

| **North Back Reef (NBR) vs. North Fore Reef (NFR): 87.25% similarity** | | | | | | |
| --- | --- | --- | --- | --- | --- | --- |
| Taxon | Mean | SD | Ratio | Mean NBR | Mean NFR | % Contribution |
| *Pseudodiploria strigosa* | 0.31 | 0.25 | 1.23 | 0.55 | <0.01 | 34.98% |
| *Porites astreoides* | 0.17 | 0.22 | 0.76 | 0.08 | 0.10 | 54.16% |
| *Orbicella annularis* | 0.08 | 0.14 | 0.57 | 0.01 | 0.06 | 63.40% |
| *Pseudodiploria clivosa* | 0.07 | 0.16 | 0.44 | 0.12 | 0.00 | 71.67% |
| *Acropora palmata* | 0.05 | 0.15 | 0.35 | 0.21 | 0.00 | 77.54% |
| *Favia fragum* | 0.05 | 0.14 | 0.32 | 0.02 | 0.00 | 82.74% |
| *Millepora* spp. | 0.04 | 0.14 | 0.30 | 0.00 | 0.04 | 87.66% |
| *Agaricia agaricites* | 0.04 | 0.10 | 0.39 | 0.01 | 0.02 | 92.21% |
| *Siderastrea siderea* | 0.04 | 0.09 | 0.39 | 0.00 | 0.04 | 96.24% |
| **North Back Reef (NBR) vs. North Reef Crest (NRC): 82.65% similarity** | | | | | | |
| Taxon | Mean | SD | Ratio | Mean NBR | Mean NRC | % Contribution |
| *Pseudodiploria strigosa* | 0.30 | 0.25 | 1.23 | 0.55 | 0.10 | 37.07% |
| *Porites astreoides* | 0.18 | 0.22 | 0.81 | 0.08 | 0.14 | 59.30% |
| *Pseudodiploria clivosa* | 0.09 | 0.17 | 0.54 | 0.12 | 0.06 | 70.28% |
| *Millepora* spp. | 0.08 | 0.25 | 0.34 | 0.00 | 0.25 | 80.50% |
| *Acropora palmata* | 0.06 | 0.14 | 0.42 | 0.21 | <0.01 | 87.90% |
| *Favia fragum* | 0.04 | 0.14 | 0.31 | 0.02 | 0.00 | 93.08% |
| *Orbicella annularis* | 0.02 | 0.06 | 0.32 | 0.01 | 0.00 | 95.44% |
| **North Back Reef (NBR) vs. South Back Reef (SBR): 75.13% similarity** | | | | | | |
| Taxon | Mean | SD | Ratio | Mean NBR | Mean SBR | % Contribution |
| *Pseudodiploria strigosa* | 0.34 | 0.25 | 1.39 | 0.55 | 0.36 | 45.88% |
| *Pseudodiploria clivosa* | 0.09 | 0.15 | 0.61 | 0.12 | 0.06 | 58.28% |
| *Porites astreoides* | 0.08 | 0.11 | 0.76 | 0.08 | 0.03 | 69.39% |
| *Orbicella annularis* | 0.06 | 0.09 | 0.66 | 0.01 | 0.08 | 77.40% |
| *Acropora palmata* | 0.05 | 0.13 | 0.35 | 0.21 | 0.00 | 83.68% |
| *Orbicella faveolata* | 0.04 | 0.12 | 0.32 | 0.00 | 0.06 | 88.64% |
| *Favia fragum* | 0.03 | 0.09 | 0.37 | 0.02 | <0.01 | 93.06% |
| *Millepora* spp. | 0.01 | 0.04 | 0.32 | 0.00 | 0.02 | 94.94% |
| *Agaricia agaricites* | 0.01 | 0.02 | 0.50 | 0.01 | <0.01 | 96.28% |
| **North Back Reef (NBR) vs. South Fore Reef (SFR): 87.95% similarity** | | | | | | |
| Taxon | Mean | SD | Ratio | Mean NBR | Mean SFR | % Contribution |
| *Orbicella annularis* | 0.28 | 0.29 | 0.96 | 0.01 | 1.04 | 31.89% |
| *Pseudodiploria strigosa* | 0.18 | 0.17 | 1.04 | 0.55 | 0.09 | 52.33% |
| *Millepora* spp. | 0.10 | 0.14 | 0.66 | 0.00 | 0.21 | 63.20% |
| *Porites astreoides* | 0.06 | 0.11 | 0.56 | 0.08 | 0.07 | 70.42% |
| *Porites porites* | 0.06 | 0.08 | 0.79 | 0.01 | 0.11 | 77.26% |
| *Pseudodiploria clivosa* | 0.05 | 0.11 | 0.40 | 0.12 | 0.00 | 82.46% |
| *Acropora palmata* | 0.04 | 0.12 | 0.34 | 0.21 | 0.00 | 87.08% |
| *Colpophyllia natans* | 0.02 | 0.07 | 0.32 | 0.00 | 0.04 | 89.60% |
| *Siderastrea siderea* | 0.02 | 0.03 | 0.86 | 0.00 | 0.05 | 92.08% |
| *Montastraea cavernosa* | 0.02 | 0.03 | 0.60 | 0.01 | 0.04 | 94.45% |
| *Favia fragum* | 0.02 | 0.06 | 0.28 | 0.02 | 0.00 | 96.30% |
| **North Back Reef (NBR) vs. South Reef Crest (SRC): 79.54% similarity** | | | | | | |
| Taxon | Mean | SD | Ratio | Mean NBR | Mean SRC | % Contribution |
| *Pseudodiploria strigosa* | 0.23 | 0.21 | 1.09 | 0.55 | 0.76 | 28.76% |
| *Acropora palmata* | 0.21 | 0.25 | 0.86 | 0.21 | 1.60 | 55.49% |
| *Millepora* spp. | 0.15 | 0.20 | 0.77 | 0.00 | 0.55 | 74.71% |
| *Porites astreoides* | 0.08 | 0.13 | 0.59 | 0.08 | 0.20 | 84.36% |
| *Pseudodiploria clivosa* | 0.06 | 0.09 | 0.68 | 0.12 | 0.12 | 92.09% |
| *Orbicella annularis* | 0.01 | 0.03 | 0.42 | 0.01 | 0.02 | 93.90% |
| *Porites porites* | 0.01 | 0.02 | 0.73 | 0.01 | 0.06 | 95.42% |
| **North Fore Reef (NFR) vs. North Reef Crest (NRC): 80.78% similarity** | | | | | | |
| Taxon | Mean | SD | Ratio | Mean NFR | Mean NRC | % Contribution |
| *Porites astreoides* | 0.28 | 0.26 | 1.07 | 0.10 | 0.14 | 34.44% |
| *Millepora* spp. | 0.15 | 0.31 | 0.49 | 0.04 | 0.25 | 53.20% |
| *Pseudodiploria strigosa* | 0.11 | 0.17 | 0.64 | <0.01 | 0.10 | 66.73% |
| *Orbicella annularis* | 0.09 | 0.16 | 0.58 | 0.06 | 0.00 | 78.14% |
| *Agaricia agaricites* | 0.04 | 0.05 | 0.83 | 0.02 | <0.01 | 83.63% |
| *Siderastrea siderea* | 0.04 | 0.11 | 0.41 | 0.04 | 0.00 | 89.11% |
| *Pseudodiploria clivosa* | 0.04 | 0.10 | 0.35 | 0.00 | 0.06 | 93.45% |
| *Acropora palmata* | 0.02 | 0.07 | 0.29 | 0.00 | <0.01 | 95.76% |
| **North Fore Reef (NFR) vs. South Back Reef (SBR): 91.21% similarity** | | | | | | |
| Taxon | Mean | SD | Ratio | Mean NFR | Mean SBR | % Contribution |
| *Pseudodiploria strigosa* | 0.41 | 0.29 | 1.39 | <0.01 | 0.36 | 44.81% |
| *Porites astreoides* | 0.13 | 0.14 | 0.95 | 0.10 | 0.03 | 58.91% |
| *Orbicella annularis* | 0.11 | 0.12 | 0.87 | 0.06 | 0.08 | 70.68% |
| *Orbicella faveolata* | 0.06 | 0.14 | 0.42 | 0.01 | 0.06 | 77.01% |
| *Pseudodiploria clivosa* | 0.06 | 0.12 | 0.47 | 0.00 | 0.06 | 83.23% |
| *Millepora* spp. | 0.06 | 0.13 | 0.43 | 0.04 | 0.02 | 89.40% |
| *Siderastrea siderea* | 0.04 | 0.08 | 0.48 | 0.04 | <0.01 | 93.83% |
| *Agaricia agaricites* | 0.03 | 0.03 | 1.02 | 0.02 | <0.01 | 96.74% |
| **North Fore Reef (NFR) vs. South Fore Reef (SFR): 85.02% similarity** | | | | | | |
| Taxon | Mean | SD | Ratio | Mean NFR | Mean SFR | % Contribution |
| *Orbicella annularis* | 0.34 | 0.31 | 1.09 | 0.06 | 1.04 | 39.45% |
| *Millepora* spp. | 0.13 | 0.17 | 0.80 | 0.04 | 0.21 | 55.10% |
| *Porites astreoides* | 0.09 | 0.15 | 0.62 | 0.10 | 0.07 | 66.11% |
| *Porites porites* | 0.08 | 0.09 | 0.85 | 0.00 | 0.11 | 75.21% |
| *Pseudodiploria strigosa* | 0.06 | 0.06 | 0.96 | <0.01 | 0.09 | 82.42% |
| *Siderastrea siderea* | 0.04 | 0.06 | 0.72 | 0.04 | 0.05 | 87.50% |
| *Colpophyllia natans* | 0.03 | 0.08 | 0.35 | 0.00 | 0.04 | 90.85% |
| *Montastraea cavernosa* | 0.02 | 0.04 | 0.57 | 0.00 | 0.04 | 93.66% |
| *Orbicella faveolata* | 0.02 | 0.04 | 0.55 | 0.01 | 0.03 | 96.01% |
| **North Fore Reef (NFR) vs. South Reef Crest (SRC): 88.37% similarity** | | | | | | |
| Taxon | Mean | SD | Ratio | Mean NFR | Mean SRC | % Contribution |
| *Pseudodiploria strigosa* | 0.24 | 0.25 | 0.97 | <0.01 | 0.76 | 27.07% |
| *Acropora palmata* | 0.21 | 0.27 | 0.79 | 0.00 | 1.60 | 50.96% |
| *Millepora* spp. | 0.19 | 0.23 | 0.84 | 0.04 | 0.55 | 72.41% |
| *Porites astreoides* | 0.10 | 0.16 | 0.63 | 0.10 | 0.20 | 83.86% |
| *Pseudodiploria clivosa* | 0.04 | 0.05 | 0.87 | 0.00 | 0.12 | 88.87% |
| *Orbicella annularis* | 0.04 | 0.06 | 0.58 | 0.06 | 0.02 | 92.87% |
| *Siderastrea siderea* | 0.02 | 0.05 | 0.38 | 0.04 | <0.01 | 95.06% |
| **North Reef Crest (NRC) vs. South Back Reef (SBR): 84.81% similarity** | | | | | | |
| Taxon | Mean | SD | Ratio | Mean NRC | Mean SBR | % Contribution |
| *Pseudodiploria strigosa* | 0.36 | 0.30 | 1.22 | 0.10 | 0.36 | 42.93% |
| *Porites astreoides* | 0.14 | 0.12 | 1.15 | 0.14 | 0.03 | 58.95% |
| *Millepora* spp. | 0.10 | 0.25 | 0.41 | 0.25 | 0.02 | 71.05% |
| *Pseudodiploria clivosa* | 0.08 | 0.13 | 0.58 | 0.06 | 0.06 | 80.01% |
| *Orbicella annularis* | 0.06 | 0.11 | 0.61 | 0.00 | 0.08 | 87.58% |
| *Orbicella faveolata* | 0.04 | 0.13 | 0.33 | 0.00 | 0.06 | 92.84% |
| *Favia fragum* | 0.01 | 0.05 | 0.28 | 0.00 | <0.01 | 94.33% |
| *Agaricia agaricites* | 0.01 | 0.02 | 0.47 | <0.01 | <0.01 | 95.52% |
| **North Reef Crest (NRC) vs. South Fore Reef (SFR): 87.88% similarity** | | | | | | |
| Taxon | Mean | SD | Ratio | Mean NRC | Mean SFR | % Contribution |
| *Orbicella annularis* | 0.32 | 0.31 | 1.03 | 0.00 | 1.04 | 36.73% |
| *Millepora* spp. | 0.17 | 0.23 | 0.76 | 0.25 | 0.02 | 56.16% |
| *Porites astreoides* | 0.09 | 0.12 | 0.75 | 0.14 | 0.07 | 66.33% |
| *Pseudodiploria strigosa* | 0.08 | 0.08 | 1.00 | 0.10 | 0.09 | 75.63% |
| *Porites porites* | 0.07 | 0.09 | 0.80 | 0.00 | 0.11 | 83.83% |
| *Colpophyllia natans* | 0.03 | 0.08 | 0.33 | 0.00 | 0.04 | 86.83% |
| *Siderastrea siderea* | 0.03 | 0.03 | 0.89 | 0.00 | 0.05 | 89.75% |
| *Pseudodiploria clivosa* | 0.02 | 0.07 | 0.33 | 0.06 | 0.00 | 92.32% |
| *Montastraea cavernosa* | 0.02 | 0.04 | 0.56 | 0.00 | 0.04 | 94.87% |
| *Orbicella faveolata* | 0.01 | 0.03 | 0.45 | 0.00 | 0.03 | 96.44% |
| **North Reef Crest (NRC) vs. South Reef Crest (SRC): 84.30% similarity** | | | | | | |
| Taxon | Mean | SD | Ratio | Mean NRC | Mean SRC | % Contribution |
| *Pseudodiploria strigosa* | 0.23 | 0.23 | 0.98 | 0.10 | 0.76 | 26.78% |
| *Millepora* spp. | 0.22 | 0.25 | 0.88 | 0.25 | 0.55 | 52.54% |
| *Acropora palmata* | 0.21 | 0.26 | 0.80 | <0.01 | 1.60 | 77.07% |
| *Porites astreoides* | 0.09 | 0.15 | 0.64 | 0.14 | 0.20 | 88.10% |
| *Pseudodiploria clivosa* | 0.05 | 0.06 | 0.84 | 0.06 | 0.12 | 94.39% |
| *Porites porites* | 0.01 | 0.02 | 0.63 | 0.00 | 0.06 | 95.74% |
| **South Back Reef (SBR) vs. South Fore Reef (SFR): 83.65% similarity** | | | | | | |
| Taxon | Mean | SD | Ratio | Mean SBR | Mean SFR | % Contribution |
| *Orbicella annularis* | 0.28 | 0.29 | 0.98 | 0.08 | 1.04 | 33.78% |
| *Pseudodiploria strigosa* | 0.18 | 0.17 | 1.06 | 0.36 | 0.09 | 55.61% |
| *Millepora* spp. | 0.10 | 0.14 | 0.70 | 0.02 | 0.21 | 67.43% |
| *Porites porites* | 0.06 | 0.07 | 0.85 | 0.01 | 0.11 | 74.73% |
| *Porites astreoides* | 0.05 | 0.09 | 0.62 | 0.03 | 0.07 | 81.10% |
| *Orbicella faveolata* | 0.04 | 0.09 | 0.44 | 0.06 | 0.03 | 85.65% |
| *Pseudodiploria clivosa* | 0.03 | 0.08 | 0.42 | 0.06 | 0.00 | 89.46% |
| *Colpophyllia natans* | 0.02 | 0.07 | 0.34 | 0.00 | 0.04 | 92.16% |
| *Siderastrea siderea* | 0.02 | 0.02 | 0.97 | <0.01 | 0.05 | 94.80% |
| *Montastraea cavernosa* | 0.02 | 0.03 | 0.62 | 0.01 | 0.04 | 97.35% |
| **South Back Reef (SBR) vs. South Reef Crest (SRC): 81.05% similarity** | | | | | | |
| Taxon | Mean | SD | Ratio | Mean SBR | Mean SRC | % Contribution |
| *Pseudodiploria strigosa* | 0.23 | 0.18 | 1.23 | 0.36 | 0.76 | 28.00% |
| *Acropora palmata* | 0.20 | 0.26 | 0.78 | 0.00 | 1.60 | 52.62% |
| *Millepora* spp. | 0.15 | 0.19 | 0.82 | 0.02 | 0.55 | 71.48% |
| *Porites astreoides* | 0.08 | 0.12 | 0.70 | 0.03 | 0.20 | 81.91% |
| *Pseudodiploria clivosa* | 0.05 | 0.06 | 0.83 | 0.06 | 0.12 | 88.14% |
| *Orbicella annularis* | 0.03 | 0.06 | 0.60 | 0.08 | 0.02 | 92.33% |
| *Orbicella faveolata* | 0.02 | 0.07 | 0.30 | 0.06 | 0.00 | 94.97% |
| *Porites porites* | 0.01 | 0.02 | 0.71 | 0.01 | 0.06 | 96.53% |
| **South Fore Reef (SFR) vs. South Reef Crest (SRC): 84.86% similarity** | | | | | | |
| Taxon | Mean | SD | Ratio | Mean SFR | Mean SRC | % Contribution |
| *Orbicella annularis* | 0.19 | 0.24 | 0.79 | 1.04 | 0.02 | 22.19% |
| *Acropora palmata* | 0.18 | 0.24 | 0.76 | 0.00 | 1.60 | 43.28% |
| *Pseudodiploria strigosa* | 0.17 | 0.16 | 1.01 | 0.09 | 0.76 | 62.82% |
| *Millepora* spp. | 0.13 | 0.15 | 0.84 | 0.21 | 0.55 | 78.01 |
| *Porites astreoides* | 0.05 | 0.07 | 0.74 | 0.07 | 0.20 | 84.11% |
| *Porites porites* | 0.04 | 0.05 | 0.74 | 0.11 | 0.06 | 88.36% |
| *Pseudodiploria clivosa* | 0.03 | 0.04 | 0.86 | 0.00 | 0.12 | 91.99% |
| *Montastraea cavernosa* | 0.01 | 0.02 | 0.55 | 0.04 | 0.01 | 93.55% |
| *Siderastrea siderea* | 0.01 | 0.02 | 0.80 | 0.05 | <0.01 | 95.08% |

**Table S5**. Summary of mean growth of *Acropora palmata* and *Pseudodiploria strigosa* at three sites around Buck Island Reef National Monument with ranges (in parentheses). The complete coral growth dataset is provided in Kuffner et al.^1^

| ***Acropora palmata*** | | | |
| --- | --- | --- | --- |
| **Site** | **Calcification rate (kg m^-2^ y^-1^)** | **Δ height (cm y^-1^)** | **Δ planar surface area (cm^2^ y^-1^)** |
| SE | 27.11  (21.04 to 34.09) | 7.95  (5.73 to 9.25) | 192.82  (105.27 to 338.56) |
| NE | 27.26  (24.66 to 28.87) | 6.69  (5.09 to 8.55) | 160.87  (51.67 to 338.40) |
| NW | 33.26  (27.61 to 42.91) | 5.88  (4.19 to 7.16) | 229.24  (95.10 to 407.80) |
| **Overall** | **29.07**  **(±1.26)** | **6.99**  **(±1.56)** | **196.21**  **(±102.95)** |
| ***Pseudodiploria strigosa*** | | | |
| **Site** | **Calcification rate (kg m^-2^ y^-1^)** | **Δ height (cm y^-1^)** | **Δ planar surface area (cm^2^ y^-1^)** |
| SE | 13.22  (8.39 to 21.31) | 0.75  (.12 to 1.72) | 8.87  (-5.00 to 18.15) |
| NE | 16.24  (8.87 to 20.93) | 1.51  (-0.50 to 2.70) | 20.15  (8.60 to 35.45) |
| NW | 15.38  (7.96 to 20.25) | 0.74  (-0.12 to 2.12) | 14.03  (-5.10 to 31.80) |
| **Overall** | **14.30**  **(±0.85)** | **0.95**  **(±0.71)** | **12.88**  **(±9.71)** |

**Table S6**. Species-specific density, linear extension, and rugosity values used in calculations of carbonate production by coral species at Buck Island Reef National Monument. Density and linear-extension values are from ReefBudget v1(ref. ^2^). The values for all species were derived from Bozec et al.^3^ except for *A. palmata*, for which we used the rugosity value determined by Alvarez-Filip et al.^4^. Asterisks indicate species for which no published values were available at the time of this study (but see Courtney et al.^5^); these values were calculated as the mean rugosity of all non-branching species in Bozec et al.^3^.

| **Taxon** | **Density**  (g cm^-3^) | **Linear extension** (cm yr^-1^) | **Rugosity** |
| --- | --- | --- | --- |
| *Acropora palmata* (branching tips) | 1.81 | 6.63 | 3.33 |
| *Acropora palmata* | 1.81 | 0.60 | 3.33 |
| *Agaricia agaricites* | 1.83 | 0.26 | 1.96 |
| *Agaricia grahamae* | 1.85 | 0.26 | 1.96 |
| *Agaricia humilis* | 1.85 | 0.26 | 1.96 |
| *Colpophyllia natans* | 0.78 | 0.99 | 2.19 |
| *Dichocoenia stokesii* | 2.30 | 0.62 | 2.19 |
| *Pseudodiploria clivosa* | 1.20 | 0.41 | 2.19 |
| *Diploria labyrinthiformis* | 1.20 | 0.34 | 2.19 |
| *Pseudodiploria strigosa* | 1.20 | 0.53 | 2.19 |
| *Favia fragum* | 1.57 | 0.73 | 2.19 |
| *Millepora* spp. | 2.27 | 1.24 | 2.19 |
| *Montastraea cavernosa* | 1.67 | 0.71 | 2.45 |
| *Orbicella annularis* | 1.58 | 0.86 | 2.39 |
| *Orbicella faveolata* | 1.39 | 0.84 | 2.80 |
| *Porites astreoides* | 1.59 | 0.45 | 1.87 |
| *Porites porites* | 1.18 | 1.34 | 2.26 |
| *Siderastrea radians* | 1.61 | 0.45 | 1.59 |
| *Siderastrea siderea* | 1.61 | 0.45 | 1.59 |
| *Stephanocoenia intersepta* | 1.57 | 0.50 | 2.19 |

**Table S7**. Bioerosion rates (g individual^-1^ day^-1^) of individual parrotfish for each species, life phase, and size class provided in ReefBudget v1 (ref.^2^). Generic-level rates were used for the six observations for which species-level identifications could not be made with confidence. The rates were determined by calculating the product of estimated bite volume, proportion of bites leaving scars, daily bite rates, and the assumed framework-density value of 1.67 cm^3^ for the western Atlantic^2^.

|  | **Initial** | |  |  | **Terminal** | |  |  |
| --- | --- | --- | --- | --- | --- | --- | --- | --- |
| **Taxon** | **5**–**14 cm** | **15**–**24 cm** | **25**–**34 cm** | **35**–**44 cm** | **15**–**24 cm** | **25**–**34 cm** | **35**–**44 cm** | **>45 cm** |
| *Scarus vetula* | 0.94 | 8.53 | 54.09 | 165.19 | 7.25 | 45.98 | 140.41 | 229.19 |
| *Scarus taeniopterus* | 0.57 | 4.71 | 26.43 | 66.85 | 4.00 | 22.47 | 56.82 | 65.93 |
| *Scarus iseri* | 0.40 | 3.05 | 14.45 | 24.26 | 2.60 | 12.29 | 20.62 | 0.00 |
| *Scarus* spp. | 0.94 | 8.53 | 54.09 | 165.19 | 7.25 | 45.98 | 140.41 | 229.19 |
| *Sparisoma viride* | 1.97 | 72.66 | 238.51 | 420.62 | 58.73 | 193.40 | 343.13 | 372.38 |
| *Sparisoma aurofrenatum* | 1.52 | 52.08 | 149.89 | 193.52 | 42.27 | 122.50 | 161.45 | 17.54 |
| *Sparisoma rubripinne* | 1.78 | 63.99 | 201.15 | 324.88 | 51.79 | 163.51 | 266.54 | 222.79 |
| *Sparisoma chrysopterum* | 1.51 | 51.68 | 148.15 | 189.06 | 41.94 | 121.11 | 157.88 | 10.58 |
| *Sparisoma* spp. | 1.97 | 72.66 | 238.51 | 420.62 | 58.73 | 193.40 | 343.13 | 372.38 |

**Table S8**. Individual bioerosion rates for sea urchins by size class based on the empirical relationship presented in ReefBudget v1 (ref. ^2^).

| **Size**  (cm) | **Erosion**  (g cm^-2^ d^-1^) |
| --- | --- |
| 0–20 | 0.02 |
| 20–40 | 0.34 |
| 40–60 | 1.18 |
| 60–80 | 2.69 |
| 80–100 | 4.99 |

**
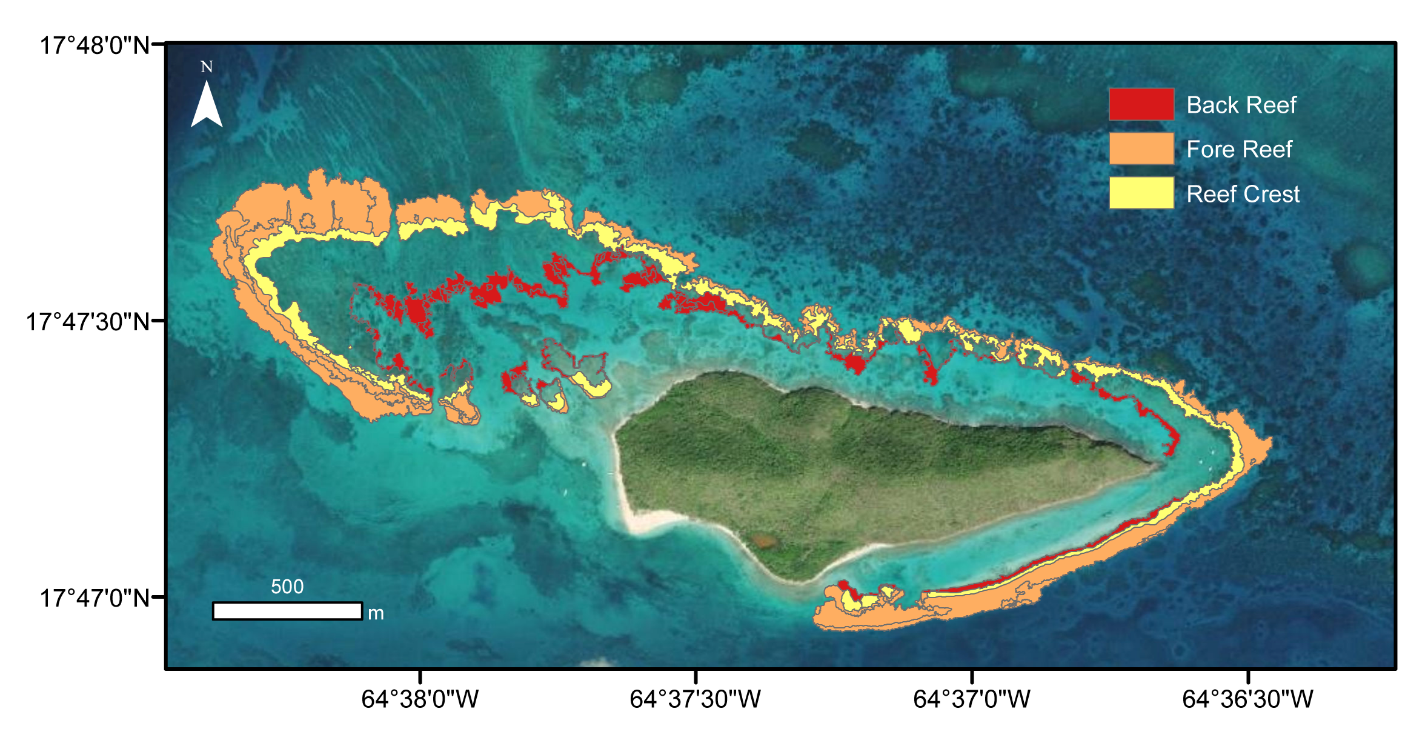
**

**Figure S1.** Map of the shallow-water coral-reef habitats at Buck Island Reef National Monument, showing the extent of the fore-reef, reef-crest, and back-reef habitats. The map was created using the benthic habitat maps generated by the National Oceanic and Atmospheric Administration (<https://products.coastalscience.noaa.gov/collections/benthic/e93stcroix/>). The 166,407 m^2^ reef-crest habitat (yellow) is where *Acropora palmata* would be outplanted in our restoration models. Map image is the intellectual property of Esri and is used herein under license. Copyright 2020 Esri and its licensors. All rights reserved.

**
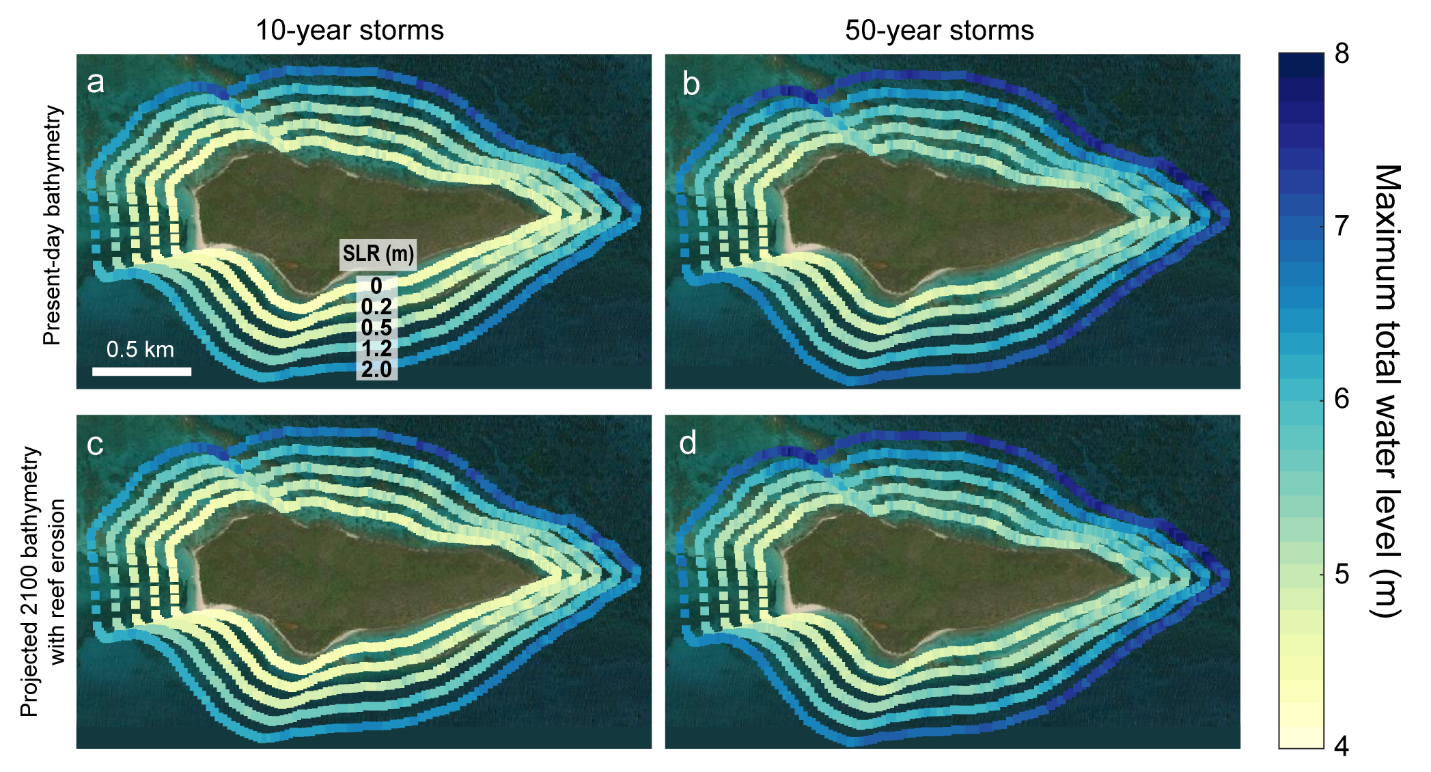
**

**Figure S2.** Maps displaying the impact of sea-level rise and coral-reef bathymetry on alongshore variation in total water levels and thus coastal-flooding potential. In each panel, the maximum total water levels at 2100 are plotted along the shoreline of the island, with each ring of points representing a sea-level rise scenario, from +0.0 m (inner ring) to +2.0 m (outer ring). Left (a & c) and right (b & d) columns present 10-year storms (i.e., a strong tropical storm or Category-1 hurricane) and 50-year storm results (i.e., a Category-5 hurricane), respectively^6^. Projected total water levels with present-day bathymetry (a & b) are compared with projected total water levels with reef erosion from 2016 to 2100 based on the carbonate-budget models (c & d; a & b in Fig. 4 in the main text). Map image is the intellectual property of Esri and is used herein under license. Copyright 2020 Esri and its licensors. All rights reserved.

**
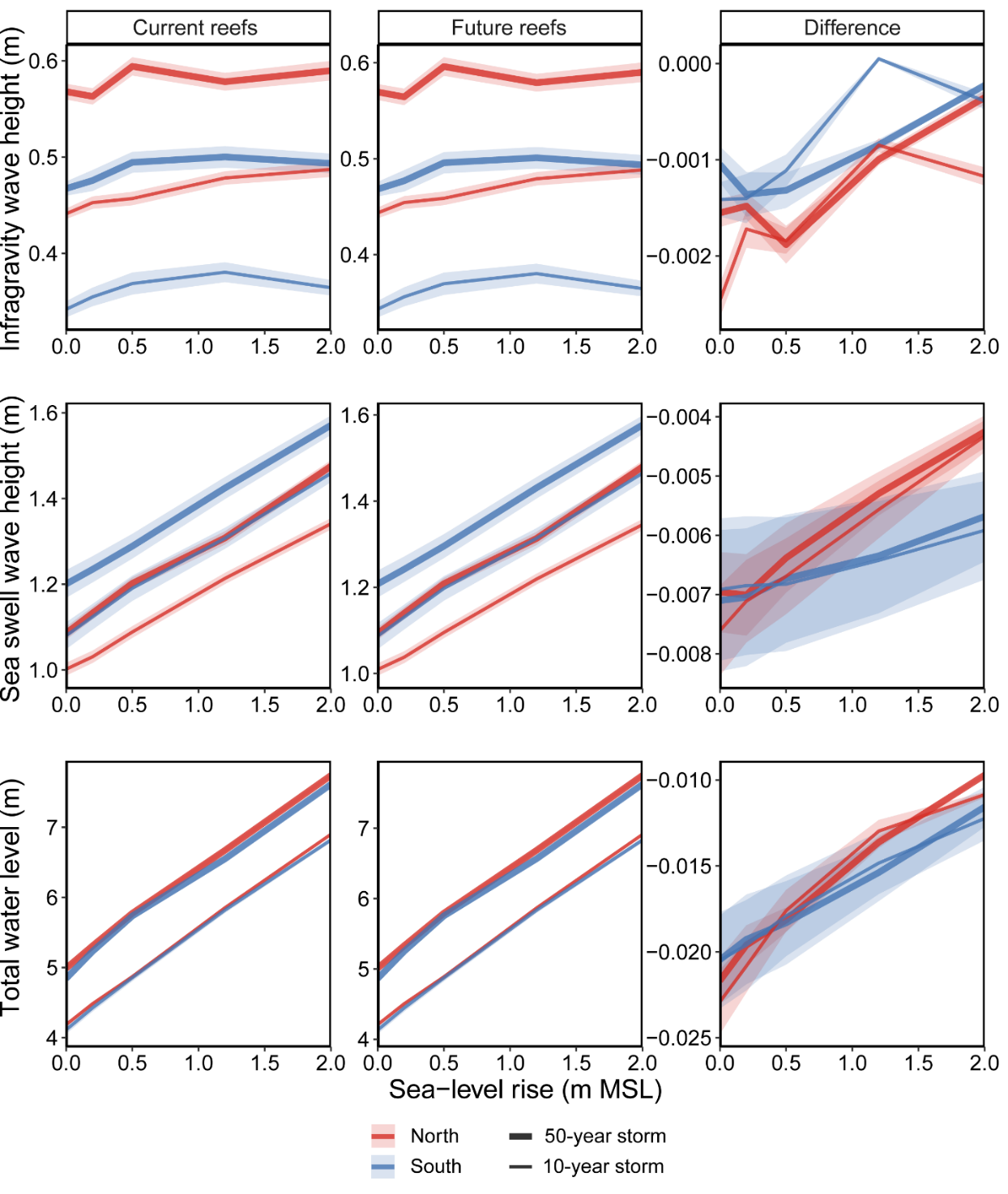
**

**Figure S3.** Plots synthesizing the impact of sea-level rise, storms, and coral-reef erosion on nearshore hydrodynamics. The eroding northern reef is denoted in red and the accreting southern reef in blue. Variation of the mean (solid lines) ± standard error (shading) response to sea-level rise (in meters relative to mean sea level [m MSL]) over all points along the shoreline for the 10-year (thin lines) and 50-year (thick lines) storm conditions. Rows are, from top to bottom, infragravity wave height (>25 s period), sea-swell wave height (5‒25 s period), and total water level. Columns are, from left to right, projections based on current reef elevation, projections based on reef elevation with reef erosion (north) or accretion (south), and the difference between the current and future reef morphology. Increasing sea level allows for larger sea swell and slightly larger infragravity waves to propagate across the reef, resulting in greater total water levels and flooding potential. The minimal trends on the south reefs relative to the north reefs presented in the right column reflect the more significant role of reef degradation in the northern sector.


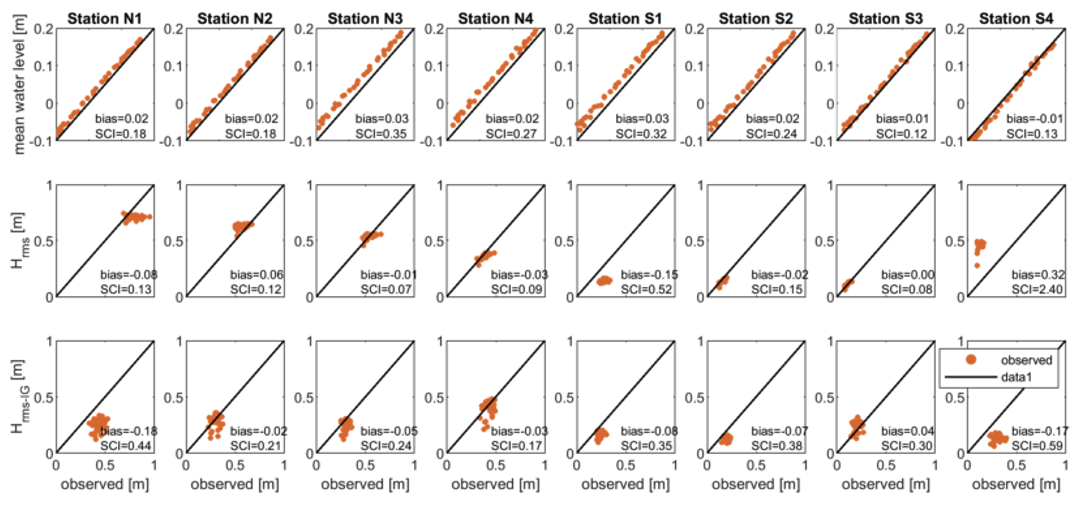


**Figure S4.** Hydrodynamic data–model comparisons, including bias and scatter-index-value metrics. The top row is mean water level (m), the middle row is root-mean-square of the sea-swell band (periods < 25 s) wave height (m), and the bottom row is root-mean-square of the infragravity band (periods > 25 s) wave height (m). The north (N) and south (S) stations are numbered from offshore (1) to onshore (4) and are plotted in Figure 1. Overall, the data–model comparisons are very good, especially under the relatively low-energy forcing conditions during the field deployments.

**Supplementary References:**

1. Kuffner, I. B. *et al.* Experimental coral-growth rates and time-series imagery data for *Acropora palmata* and *Pseudodiploria strigosa* in St. Croix, U.S. Virgin Islands. *U.S. Geological Survey data release*, https://doi.org/10.5066/P5094BOI5069T (2022).

2. Perry, C. T. *et al.* ReefBudget: Methodolgy. http://geography.exeter.ac.uk/reefbudget/ (2012).

3. Bozec, Y.-M., Alvarez-Filip, L. & Mumby, P. J. The dynamics of architectural complexity on coral reefs under climate change. *Global Change Biology* **21**, 223-235, doi:https://doi.org/10.1111/gcb.12698 (2015).

4. Alvarez-Filip, L., Carricart-Ganivet, J. P., Horta-Puga, G. & Iglesias-Prieto, R. Shifts in coral-assemblage composition do not ensure persistence of reef functionality. *Scientific Reports* **3**, srep03486 (2013).

5. Courtney, T. A. *et al.* Area-normalized scaling of ReefBudget calcification, macrobioerosion, and microbioerosion rates for use with CoralNet Version 1.0 https://doi.org/10.5281/zenodo.5140477 (2021).

6. Parisi, F. & Lund, R. Return Periods of Continental U.S. Hurricanes. *Journal of Climate* **21**, 403-410, doi:10.1175/2007jcli1772.1 (2008).
